# Effect of Training Program on Evidence-Based Practice Competencies in Hospital Nurses in Taiwan: A Quasi-Experimental Study

**DOI:** 10.1097/jnr.0000000000000701

**Published:** 2025-09-12

**Authors:** Mei-Ling YEH, Bieng-Yi CHANG, Hung-Da DAI, Min-Huey CHUNG

**Affiliations:** 1 Department of Nursing, School of Nursing, National Taipei University of Nursing and Health Sciences, Taipei City, Taiwan; 2 Department of Nursing, Cheng Hsin General Hospital, Taipei City, Taiwan; 3 Department of Nursing, Taipei Veterans General Hospital, Taipei City, Taiwan; 4 Department of Nursing, School of Nursing, College of Nursing, Taipei Medical University, Taipei City, Taiwan; 5 Department of Nursing, Shuang Ho Hospital, Taipei Medical University, New Taipei City, Taiwan

**Keywords:** competency, evidence-based practice, nurse, training program

## Abstract

**Background:**

Evidence-based practice has been shown to improve the quality and efficiency of health care. However, more than 58% of nurses in Taiwan have no evidence-based-practice-related training program experience.

**Purpose:**

This study was designed to establish a training program to improve the evidence-based practice competencies among currently practicing clinical nurses.

**Methods:**

A pretest–posttest quasi-experimental study was conducted on a sample of 80 clinical nurses aged 20 and above. The sample was distinguished into exposure (training program) and control groups. The Health Sciences-Evidence-Based Practice questionnaire was used to measure targeted outcomes before and after the training program. IBM SPSS version 20.0 was used to perform k-means cluster analysis, with *p* values <.05 considered statistically significant.

**Results:**

After the training program intervention, statistically significant between-group differences were found in the five domains of evidence-based practice, including belief attitudes, results of scientific research, development of professional practice, results assessment, and barriers-facilitators for evidence-based practice. The k-means of cluster analysis resulted in the regrouping of the exposure group with the EBP program as exposure A (*n*=20) and exposure B (*n*=20). Significant differences were observed for all five domains of the Health Sciences-Evidence-Based Practice among the three groups, with scores for the exposure A group consistently better than those for either the exposure B or control group, especially in the results of scientific research and results assessment domains.

**Conclusions:**

The 40-hour evidence-based practice training program is an effective program for improving evidence-based practice competencies in health care professionals. This training program may be applied as a tool to help clinical nurses correctly and effectively apply evidence-based practice in patient health care.

## Introduction

Effective and satisfactory health outcomes are necessarily associated with increased spending on health care (Baicker & Chandra, 2004). Evidence-based practice (EBP) has been recommended as an effective approach to improving care quality and efficiency (Fu et al., 2020) and has been identified as crucial to improving patient care in general (Kazdin, 2008). Incorporating EBP in health care system standards, policies, job descriptions, performance evaluations, and medical ladder promotion processes can drive higher health care quality and efficiency and reduce related costs (Melnyk et al., 2014; Mendagudli, 2022). Implementing EBP has been shown to improve care quality, patient safety, treatment outcomes, and health care costs and to promote high-value health care (Tucker et al., 2021). Despite the numerous recognized benefits of EBP, it has yet to be embraced globally as part of the general standard of care. EBP is a well-accepted health care model for nursing practice (Chang et al., 2010; Ward et al., 2021), and related training programs are used in Taiwan to provide nurses with relevant knowledge and skills and facilitate its dissemination throughout the clinical practice environment (Weng et al., 2015). Moreover, health care professionals are expected to gain competencies in EBP-related skills and attitudes during their professional training (Amit-Aharon et al., 2020). Numerous studies have evaluated the effectiveness of EBP education in clinical nurses (Hsieh & Chen, 2020; Park et al., 2020). Online and face-to-face intervention methods have been the most frequently used methods (Chu et al., 2019; Mears & Blake, 2017; Moore, 2017), while computer-based learning models, self-directed learning, and mentorship programs have also been used (Spiva et al., 2017). Generally, EBP training for nurses has centered on the five steps of ask, acquire, appraise, apply, and assess (the “Five As” Melnyk et al., 2012). Also, the “patient/problem, intervention, comparison, outcome” (PICO) format has been an essential component in related training.

The main role of medical institutions in this context has been to support the implementation and application of evidence-based research and promote the translation of evidence-based knowledge. Nursing professionals around the world are actively engaged in clinical education and EBP implementation (Mackey & Bassendowski, 2017). However, more than half (an estimated 58%) of nurses in Taiwan have never attended EBP-related training (Hsu et al., 2015). The three main obstacles in promoting EBP are lack of confidence in EBP skills; difficulties with understanding, interpreting, and applying published research; and time constraints (Tsai et al., 2010). A considerable amount of uncertainty exists in exactly what evidence-based practice means. Also, lack of clarity on EBP standards and specific competencies hinders hospitals from delivering evidence-based, high-value, and low-cost health care (Duncombe, 2018). Although the effectiveness of EBP training for clinical nurses has been evaluated in several studies (e.g., Cheng, 2003), obstacles to promoting and implementing EBP in practice and inadequate training designs may result in unsatisfactory outcomes (Mears & Blake, 2017; Moore, 2017). Moreover, some of the related studies did not include a control group (Spiva et al., 2017; van der Goot et al., 2018), while others failed to use instruments with psychometric validity to measure EBP competence (Higuchi et al., 2015). Furthermore, only a few related studies have been conducted in Taiwan (Chu et al., 2019). Establishing clearly articulated EBP competencies will be critical to promoting and establishing EBP within health care organizations as a standard procedure. Based on the above, this study was developed to design a training program to establish and improve EBP competencies in clinical nurses and to evaluate the effectiveness of this program on a sample of clinical nurses in Taiwan.

## Methods

### Design

This single-blinded (assessor-blinded) pretest–posttest quasi-experimental study was conducted between May 2018 and July 2019. The experimental group received a 40-hour training program, while the control group did not receive any intervention. EBP competence was evaluated using the Health Sciences-Evidence-Based Practice (HS-EBP) questionnaire, which was administered by an assessor before and after the program. The assessor was blinded to group assignment. The EBP competence of participants was followed up 1 year after the training program by another assessor.

### Setting and Participants

Inclusion criteria were clinical nurses who were over 20 years old and had been working full-time in one or more hospitals for at least 3 months. Nurses working in an administrative capacity were excluded from participation. The minimum sample size was estimated to be 52 using G*Power 3.1 software with a significance level of .05, effect size of 0.20 (Kim et al., 2019), and power of 80%. To compensate for potential dropouts, a target number of 80 was enrolled. A convenience sampling method was used to recruit 40 clinical nurses for the exposure group from one regional teaching hospital in Taiwan. The exposure group was further subdivided into exposure A (*n*=20) and exposure B (*n*=20) groups as a consequence of the cluster analysis k-means algorithm. A further 40 clinical nurses were recruited and enrolled in the control group by posting a recruitment flyer at a district hospital. Ward/unit, age, educational level, nursing clinical ladder level, years of nursing work experience, and evidence-based nursing program experience were statistically similar in the exposure and control groups.

### Interventions

**Figure 1:**
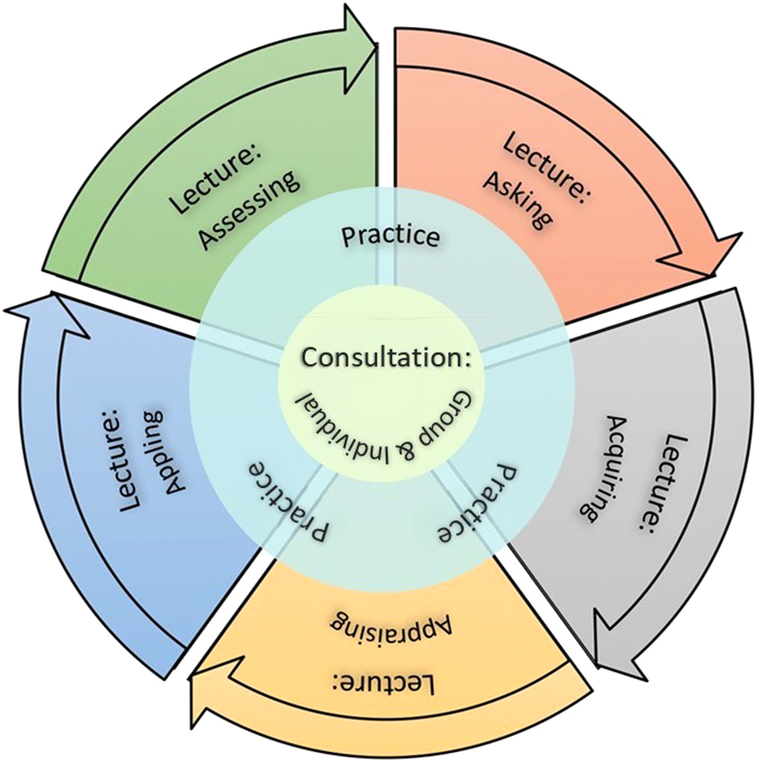
Main Context of Evidence-Based Practice Training Program *Note.* Content=The main content of the lecture includes the critical steps of the evidence-based practice (EBP) process consisting of 5As (ask, acquire, appraise, apply, and assess); Lecturer=The instructor should have a nursing, medical, and statistics background as well as expertise in empirical research; Teaching Method=Face-to-face teaching method is adopted

The design of the 40-hour EBP training program, illustrated in Figure 1, is based on the lecture, practice mentorship, and characteristics of the participants and participating institutions. EBP in the context of nursing is generally based on the five steps of ask, acquire, appraise, apply, and assess (i.e., the “Five As”). Another essential component of EBP training is learning the skills needed to apply the patient/problem, intervention, comparison, outcome (PICO) format. Also, face-to-face methods, self-directed learning, and mentorship programs (Spiva et al., 2017) are widely used.

As shown in Table 1, the exposure group participated in a 40-hour EBP training program that included 20 hours of face-to-face lectures, 10 hours of group and individual consulting, and 10 hours of hands-on training and participation in evidence-based campaigns and nursing conferences. This program was delivered over 2 weeks, after which the posttest questionnaire was administered. The exposure group comprised four teams, with each team mentored by a well-trained, evidence-based nursing facilitator.

**Table 1:** The Activities for Each Lecture, Practice, and Consultation

Item	Activity
Lecture (20 hr)	1. A self-reflection activity based on each subject is conducted by facilitators and teaching assistants of each group synchronously. 2. Debriefing meetings with facilitators and teaching assistants are conducted after each class.
Practice (10 hr)	1. The most common medical databases, such as PubMed, Cochrane Library, and Medline, are searched, and the meta-analysis results are converged and interpreted. 2. Publishing an experience in a systematic review and meta-analysis is necessary for instructors. 3. Hands-on training in groups. 4. At the end of each course, facilitators and teaching assistants guide the participants to complete the research and interpretation of the article. 5. Debriefing meetings with facilitators and teaching assistants after each class is conducted
Consultation (10 hr)	1. Reinforcement of lectures and practices, Q&A section, and counseling for writing an EBP article. 2. Practice in groups and individually. 3. Each facilitator provides consultation by appointment to 10 participants of a group. Participants participating in evidence-based campaigns and EBP-related conferences receive consultation, discussion, and guidance at the individual level.

### Instruments

Demographic information collected in this study included age, gender, educational level, marital status, working unit, years of nursing work experience, nursing clinical ladder level (N, N1, N2, N3, and N4), and evidence-based nursing activity experience.

EBP outcome was measured using the HS-EBP (Health Sciences-Evidence Based Practice) questionnaire (Fernández-Domínguez et al., 2017), which was designed to assess EBP performance and the factors influencing EBP performance improvement. EBP provides to health science professionals a standardized procedure for responding to most clinical situations arising in daily practice, and this tool is useful in both assessing individual EBP aptitude and evaluating the effectiveness of interventions designed to improve EBP performance. The HS-EBP encompasses 60 items in the five domains of beliefs-attitudes (BeAt, 12 items), results from scientific research (Science, 14 items), development of professional practice (Practice, 10 items), assessment of results (Assess, 12 items), and barriers-facilitators for EBP (BaFa, 12 items). A Likert scale, ranging from 1 to 10, is used to score all items, with higher scores indicating a greater degree of agreement. The Chinese version of the HS-EBP (Yeh et al., 2018) was used in this study. The Cronbach αs of internal consistency reliability for the BeAt, Science, Practice, Assess, and BaFa subscales were .93, .96, .84, .94, and .91, respectively, in Fernández-Domínguez et al. (2017) and .98, .97, .95, .97, and .96, respectively, in this study. Details regarding participant participation and/or achievement in EBP-related campaigns and conference submissions were recorded for one full year after the conclusion of the training program.

### Data Analysis

All statistical analyses were performed using IBM SPSS Statistics 20.0 (IBM Corp., Armonk, NY, USA), with demographic and clinical data presented as descriptive statistics. To verify homogeneity between groups, the χ^2^ test was used for categorical data and the independent *t* test was used for continuous data. To differentially identify the effects of the training program on the EBP performance of individual participants, HS-EBP data were further analyzed using an independent *t* test and k-means cluster analysis. The number of final clusters was determined using automatized methods, and no predefined number of clusters was set. Furthermore, a one-way analysis of variance and then a Scheffe test were performed to analyze the intervention effect among the groups. Finally, a linear random-effects model was used to analyze the change within each group. For all analyses, a *p* value of <.05 was considered statistically significant. In the pretest, as some in the exposure group were identified as being relatively more competent in EBP, this group was further divided into exposure A (*n*=20) and exposure B (*n*=20) groups with a high cluster quality (0.7) for subsequent data analysis work. Participants in the exposure A group had prior participation experience in evidence-based campaigns, with one earning a bronze medal and two invited to present at the International Council of Nurses oral section.

### Ethical Considerations

This study was approved by the institutional review board of Cheng-Hsin General Hospital under CHGH-IRB No: (627) 106-35, as well as by the administrators of the nursing department. Written informed consent was signed by all of the participants. All of the participants enrolled voluntarily after being provided a detailed explanation regarding the purpose and procedures of this study. Confidentiality was maintained by assigning code numbers (rather than names) to each answer sheet. All of the participants were made aware that the data collected would be kept confidential by the researcher and that they could withdraw from the study at any time.

## Results

A total of 80 participants (40 in each group) were recruited. The demographic characteristics and HS-EBP data of all participants at baseline are shown in Table 2. The mean (*SD*) ages of participants in the exposure and control groups were 37.60 (8.32) and 37.03 (7.82) years, respectively, and their average years of nursing work experience were 14.25 (8.20) and 14.55 (7.19) years, respectively. Most of the participants held bachelor's degrees, were at the N2 level of the nursing clinical ladder, and were currently working in intensive care units. In terms of beliefs and attitudes, the mean score (64.23, *SD*=19.27) for the exposure group was higher than the control group (58.56, *SD*=16.44). Similarly, the mean score for queries focusing on the development of professional practice was slightly higher in the exposure group (64.23, *SD*=17.45) than the control group (63.23, *SD*=14.35). Conversely, the control group had higher mean scores for items related to the assessment of results (63.52 vs. 61.13, *SD*=16.32 vs. 16.78) and barriers-facilitators of evidence-based practice (56.04 vs. 52.69, *SD*=13.53 vs. 19.93). In terms of items related to the results of scientific research, the mean scores measured for the exposure and control groups were similar (54.09, *SD*=18.96 and 54.45, *SD*=14.71, respectively). After the training program, an independent *t* test was used to assess intergroup differences, resulting in statistically significant differences in the BeAt (*t*=7.47, *p*<.001), Science (*t*=6.49, *p*<.001), Practice (*t*=4.74, *p*<.001), Assess (*t*=3.94, *p*<.001), and BaFa (*t*=3.45, *p*=.001) domains. Furthermore, the k-means of cluster analysis were based on the pretest/posttest change in scores for all five domains of HS-EBP. Therefore, the exposure group was further divided into exposure A (*n*=20) and exposure B (*n*=20) groups with a high cluster quality (0.7) for subsequent data analysis, as exposure group B had the highest pretest scores in all five domains of the HS-EBP. Moreover, with regard to the exposure B group, 100% of participants had previously participated in evidence-based campaigns.

**Table 2:** Demographic Characteristics and Health Sciences-Evidence Based Practice Scores of Participants at Baseline

Variable	*n* (%)		χ^2^ (*p*)
	Exposure (*n*=40)	Control (*n*=40)	
Gender			2.37 (.12) ^a^
Female	36 (90.0)	40 (100)	
Male	4 (10.0)	0 (0.0)	
Educational level			0.44 (.80)
Diploma degree	5 (12.5)	6 (15.0)	
Bachelor degree	28 (70.0)	29 (72.5)	
Master degree	7 (17.5)	5 (12.5)	
Nursing clinical ladder level ^b^			1.46 (.83)
N	5 (12.8)	3 (7.5)	
N1	3 (7.7)	5 (12.5)	
N2	14 (35.9)	17 (42.5)	
N3	13 (33.3)	12 (30.0)	
N4	4 (10.3)	3 (7.5)	
Experience with evidence-based nursing activities			0.05 (.66)
Yes	19 (47.5)	21 (52.5)	
No	21 (52.5)	19 (47.5)	
Working ward/unit			
Medical	5 (12.5)	5 (12.5)	
Surgical	5 (12.5)	5 (12.5)	
Integrated	9 (22.5)	9 (22.5)	
Cardiovascular	4 (10.0)	4 (10.0)	
Gynecological	1 (2.5)	1 (2.5)	
Psychiatric	2 (5.0)	2 (5.0)	
Intensive care	12 (30.0)	12 (30.0)	
Emergency	2 (5.0)	2 (5.0)	
Variable	Mean (*SD*)		*t* (*p*)
Age (year)	37.60 (8.32)	37.03 (7.82)	0.32 (.75)
Years of nursing work experience	14.25 (8.20)	14.55 (7.19)	−0.17 (.86)
Beliefs-attitudes (BeAt)	64.23 (19.27)	58.56 (16.44)	1.41 (.16)
Results from scientific research (Science)	54.09 (18.96)	54.45 (14.71)	−0.09 (.93)
Development of professional practice (Practice)	64.23 (17.45)	63.23 (14.35)	0.28 (.78)
Assessment of results (Assess)	61.13 (16.78)	63.52 (16.32)	−0.65 (.52)
Barriers-facilitators for Evidence-Based Practice (BaFa)	52.69 (19.93)	56.04 (13.53)	−0.88 (.38)

^a^ Fisher’s exact test; ^b^ Missing data (*n* =1).

The analysis results showing the effect of the training program on EBP performance are presented in Table 2. The exposure A group earned lower mean scores in the BeAt, Practice, and BaFa dimensions at posttest than the exposure B group. A significant difference between pretest and posttest was observed in terms of the change in scores in all five domains of the HS-EBP questionnaire among the three groups, with the exposure A group performing better than the exposure B and control groups (*p*<.05). A linear random-effects model was used to analyze heterogeneity within each group. The exposure A group showed improved performance in all five domains, with the most improvement shown in Science (mean difference [MD]=29.93; 95% CI [20.54, 39.32]), followed by Assess (MD=25.04; 95% CI [16.63, 33.45]). The exposure B group presented a significant improvement in the BeAt dimension (MD=6.30; 95% CI [−0.80, 13.40]), while the control group showed a decline in the Science dimension (MD=−0.86; 95% CI [−6.88, 5.16]).

## Discussion

The effects of a 40-hour training program on EBP performance in nurses were investigated in this study. The well-designed and diverse approach to learning used in this program facilitated the acquisition of basic knowledge through classroom lectures and practical exercises and of more in-depth knowledge through facilitator consultations. A quasi-experimental study design was used to compare EBP performance between the exposure and control groups before and after the intervention, while a validated tool was used to assess EBP competency. The results revealed that the 40-hour training program improved EBP competencies significantly. In this training program, the facilitators not only guide participants to complete research article searches and interpretations but also facilitate participants to solve technical difficulties encountered in actual practice. In light of previous findings showing that mentorship programs represent an important tool in promoting EBP-related beliefs, knowledge, and acutalization (Alexander et al., 2022; Patton et al., 2022; Spiva et al., 2017), mentors and facilitators may be essential to designing EBP training programs.

**Figure 2:**
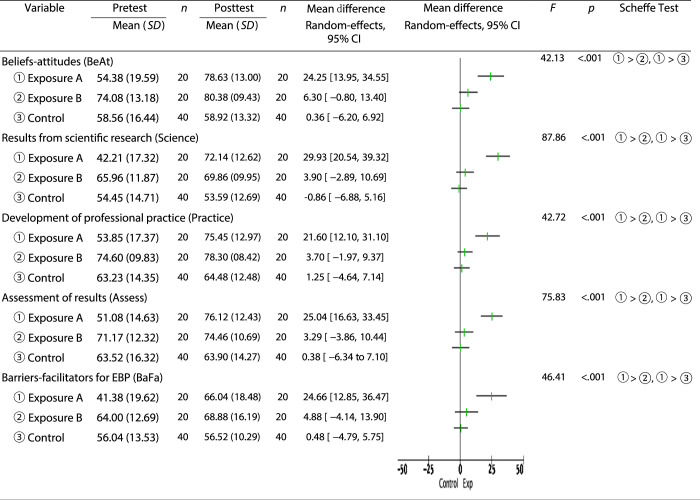
Effect of the Training Program on Evidence-Based Practice (EBP) Competencies

Despite the improved competencies in all EBP domains achieved in the exposure group, several areas were identified that may not be sufficiently addressed in the training program. For example, even with posttest improvements, barriers-facilitators for EBP (BaFa) scores in both groups were <70 (Figure 2). This may be because the work environment impediments differed among workplaces. Although a prior study reported that perceived EBP-related barriers for nurses are similar across workplaces (Alatawi et al., 2020; van der Goot et al., 2018), EBP education interventions can still assist in overcoming barriers in the work environment (Horntvedt et al., 2018). Therefore, improving structural support using training courses may require more comprehensive policy implementation within organizations.

Pretest EBP scores differed between the exposure A and exposure B groups, with mean EBP competency scores for all five domains lower in exposure A than in exposure B. At posttest, the exposure A group had improved significantly in each domain, especially Science and Assess (Figure 2). In the HS-EBP (Fernández-Domínguez et al., 2017), science refers to the competency of asking questions and evaluating the results of scientific applications in clinical practice, while assess refers to knowledge related to measuring results and analyzing data for decision-making. These abilities correspond to the 5As of the EBP steps and may be strengthened through the developed training program. Prior research results indicate education programs are an effective method of enhancing knowledge and skills in nurses (Chu et al., 2019; van der Goot et al., 2018). Thus, training programs such as that proposed in this study have the potential to significantly improve EBP competency among nurses without prior experience or knowledge of EBP.

The exposure B group earned higher scores in all five HS-EBP domains than either the exposure A or the control group at pretest, implying that exposure B group participants already had a certain level of knowledge regarding EBP. Although exposure B group mean scores did not increase significantly after training, they did show improvement, particularly in the BeAt dimension (Figure 2), indicating that the perceived importance of EBP was enhanced in these nurses after the training program, resulting in increased willingness to participate in EBP-related activities (Fernández-Domínguez et al., 2017). In addition, some studies have identified a positive effect of training programs on nurse attitudes and beliefs toward performing EBP (Chu et al., 2019). Based on these findings, nurses who already have a certain level of EBP knowledge may be expected to benefit from continued training in terms of further strengthening their motivation and willingness to participate in EBP.

### Limitations

Several limitations of this study should be considered. First, a convenience sample recruited from one hospital was used, which may introduce sampling bias. Second, group allocation was not randomized but was rather based on participant intentions. Thus, exposure group participants may have had more EBP-related interest and willingness than control group participants before study enrollment. However, as shown in Table 2, the HS-EBP domain scores did not differ significantly between the two groups at baseline. Third, other factors that may also influence EBP competency in nurses such as workplace culture and professional values (Skela-Savič et al., 2017) were not investigated in this study.

### Conclusions

The findings of this study highlight the effects of a 40-hour training program on EBP performance in nurses. Postintervention BeAt, Science, Practice, Assess, and BaFa domain scores improved significantly more in the exposure A group than in either the exposure B or control group. Furthermore, the results of a cluster analysis indicate clinical nurses with higher levels of prior EBP knowledge can still benefit from training, especially in the competency domains of science and assess. Importantly, continuous EBP education and organizational support will be crucial to effectively performing EBP in clinical workplaces.

### Implication for Nursing Management

EBP recognizes the importance of taking patient preferences, values, and feedback prudently into consideration, and encourages patients to take part in clinical therapeutic decision-making. Clinical nurses must have the competencies to have the credibility and capacity necessary to implement EBP effectively in patient health care. The EBP training program developed in this study was successful in achieving its primary goal of developing EBP competencies in participants through learning and practice.

### Clinical Resources

Evidence-based practice: https://www.nurse.com/evidence-based-practice; evidence-based practice competencies: https://fuld.nursing.osu.edu/
